# Natural Infection of Pregnant Cows with Schmallenberg Virus – A Follow-Up Study

**DOI:** 10.1371/journal.pone.0098223

**Published:** 2014-05-22

**Authors:** Kerstin Wernike, Mark Holsteg, Horst Schirrmeier, Bernd Hoffmann, Martin Beer

**Affiliations:** 1 Institute of Diagnostic Virology, Friedrich-Loeffler-Institut (FLI), Greifswald - Insel Riems, Germany; 2 Bovine Health Service, Chamber of Agriculture for North Rhine-Westphalia, Bonn, Germany; University of Liverpool, United Kingdom

## Abstract

Schmallenberg virus (SBV), an orthobunyavirus discovered in European livestock in late 2011 for the first time, causes premature or stillbirth and severe fetal malformation when cows and ewes are infected during pregnancy. Therefore, cattle of two holdings in the initially most affected area in Germany were closely monitored to describe the consequence for fetuses and newborn calves. Seventy-one calves whose mothers were naturally infected during the first five months of pregnancy were clinically, virologically, and serologically examined. One calve showed typical malformation, another one, born without visible abnormalities, was dead. Two cows aborted during the studied period; spleen and brain samples or meconium swabs were tested by real-time PCR, in none of the fetuses SBV-specific RNA was detectable and the tested fetal sera were negative in a commercially available antibody ELISA. In contrast, in nine clinically healthy calves high SBV-antibody titers were measurable before colostrum intake, and in meconium swabs of six of these animals viral RNA was present as well. The mothers of all nine seropositive calves were presumably infected between days 47 and 162 of gestation, which is within the critical timeframe for fetal infection suggested for SBV and related viruses.

## Introduction

Schmallenberg virus (SBV), an insect-transmitted orthobunyavirus that infects ruminants emerged at the German-Dutch border in late 2011, and thereafter spread rapidly within European livestock [1], [2]. In spring 2012 a high seroprevalence of about 70 to nearly 100% was found in cattle in the 2011 most affected region (German-Dutch-Belgian border) [3]–[5].

Affected adult ruminants show none or only mild clinical signs and milk drop for a few days, but an infection during a vulnerable gestation period may lead to premature or stillbirth and the birth of severe malformed calves and lambs. Related to the cattle population of a country, the rate of virus transmission from the dam to the fetus and a subsequent induction of malformation seems low [6]. In the present study, the ratio of animals infected during gestation and a vertical transmission was assessed for an individual farm. In addition to the impact on farm level, the exact gestation period for fetal SBV-infection and the induction of miscarriage or the birth of malformed calves is unknown. During an infection of the dam in the first month of pregnancy with Akabane virus (AKAV), another teratogenic virus closely related to SBV, the virus is usually not transferred to the embryo. Later on in gestation AKAV may cause varying degrees of fetal malformation primarily in the central nervous system and skeletal muscle [7]. The critical phase for cattle is most probably between the 3^rd^ and 6^th^ month of the nine-month gestation [7]–[9]. However, to verify this assumption for SBV-infection too, calves whose mothers were naturally infected with SBV between days 13 and 162 of pregnancy were monitored in two farms in Germany.

## Materials and Methods

### Cattle farms

Two private dairy cattle holdings were monitored; both farms are located in the federal state North Rhine-Westphalia (46459 Rees and 46534 Dinslaken), Germany, in the core region of the 2011 SBV-epidemic. On farm A, the approximately 130 lactating cows (Holstein-Friesian) were kept indoors; the milk yield per cow and year is about 10 500 kg. On holding B, 44 lactating Holstein-Friesian cows were kept in September 2011 in a combination of stable and pasture; the annual milk yield per animal is about 9 800 kg. Robotic milking systems were used on both farms. On both farms only diary cows were kept; the study did not involve endangered or protected species.

Starting in early September 2011 fever and a significantly reduced milk yield were reported from holding A. Whole blood and serum samples taken from 8 dairy cattle on 07^th^ of September were tested by an SBV L-segment specific real-time RT-PCR [2]. One cow sampled during the phase of decrease in milk production tested positive (serum quantification cycle value (Cq): 26.3, whole blood Cq: 27.2). Seven serum samples were examined by a commercially available SBV antibody ELISA (ID Screen Schmallenberg virus Indirect, IDvet, France), all of them tested negative.

In mid of September similar clinical symptoms were reported from holding B, in addition some cows showed diarrhea for several days. On 16^th^ of September from 5 diary cattle whole blood samples were taken, two samples scored positive. One of them was obtained from a cow with diarrhea (Cq: 39.9), the second one from an animal showing recurrent fever (Cq: 27.0). The whole blood samples were analyzed by a commercially available SBV antibody ELISA, four of them tested negative, the fifth positive.

As both farms were among the first holdings affected by SBV in 2011, the course of each pregnancy and the newborns were monitored. Under the control of the Bovine Health Service (Chamber of Agriculture for North Rhine-Westphalia) samples were taken by the responsible farm veterinarian in the context of the health monitoring of the farms. During the curative veterinary health program, a single blood sample per animal was taken by jugular venipuncture and meconium samples non-invasively by swabs; no specific permissions were required for the curative routine sampling for diagnostics by the respective farm veterinarians.

### Sampling, serology and real-time RT-PCR

In December 2011 serum samples taken from 186 cows (holding A 136 and holding B 50 samples) were tested in an indirect immunofluorescence test using SBV-infected BHK-21, clone BRS5 cells (L194, Collection of Cell Lines in veterinary Medicine, Insel Riems) as antigen matrix [10].

The births were monitored in holding A between the 27^th^ of January and 30^th^ of May and between the 16^th^ of January and 10^th^ of June in holding B, the calves were clinically examined by a veterinarian immediately after birth, meconium swabs were tested by an SBV S-segment specific real-time RT-PCR [11], and pre-colostral serum samples and sera taken from the dams were examined by a commercially available SBV antibody ELISA (ID Screen Schmallenberg virus Indirect, IDvet, France). The calves were monitored by the respective farmer for 14 days. In total, 73 calves were born and 71 were included in the present study (table 1), for the remaining two calves colostrum intake before sampling could not be excluded.

**Table 1:** Summary of calves borne in both farms in North Rhine-Westphalia, Germany.

Day of infection during gestation	Number of calves born dead/aborted	Number of calves born healthy	Number of seropositive healthy calves	number of viral RNA positive healthy calves
0–46	0	10 (A: 8/B: 2)	0	0
47–107	4 (A: 2/B: 2)	32 (A: 27/B: 5)	6 (A: 5/B: 1)	3 (A: 3/B: 0)
108–136	0	17 (A: 16/B: 1)	0	0
137–162	1 (A: 0/B: 1)	7 (A: 3/B: 4)	3 (A: 1/B: 2)	3 (A: 1/B: 2)

The first number indicates the total number of calves in the respective group, inside the parentheses the number of calves is given separately for both holdings.

## Results

### Adult animals

149 out of 186 (80.1%) serum samples taken in December 2011 were positive in the indirect immunofluorescence test, 15 doubtful (8.1%), and 22 (11.8%) negative.

In total, 70 postpartal serum samples (73 borne calves, two cases of twin pregnancy, one mother not tested) were analyzed, 66 of them tested positive by ELISA, only the mothers of calves no. 10, 12, 17, and 32 were seronegative (4 out of 70 = 5.7%); the mother of calve 68 was not tested. 20 out of 70 cows whose postpartal sera were tested were included in the sampling in December 2011, in 19 of them SBV-specific antibodies were detectable. The mother of calf no. 32 was seronegative.

The present study acts on the assumption that all animals were infected nearly simultaneously, and this time point is defined as the day of SBV-genome detection in one (holding A) or two (holding B) blood samples taken at the acute phase of the disease in adult animals. Consequently, all animals of holding A and B, respectively, are considered to be infected on 07^th^ or 16^th^ of September. Assuming the beginning of pregnancy at the day of artificial insemination, the dams were infected between gestation days 13 and 141 (holding A) or between days 16 and 162 (holding B).

### Fetuses and newborns

During the observed period two cows aborted at days 190 and 250, respectively. In the first case the dam was presumably infected with SBV after 60 days of gestation, but no pathomorphological abnormalities were visible in the fetus; during histological examination of the central nervous system no indication to a previous SBV-infection were found, and spleen, cerebrum and cerebellum tested negative by real-time RT-PCR, serum was not available in this case. The second cow (twin pregnancy) was presumably infected at day 100 and aborted at day 250 of gestation; in both fetuses neither SBV-specific antibodies nor viral RNA were detectable.

Another calf has been born at term, but dead, and spleen, cerebrum and cerebellum tested negative by real-time RT-PCR, serum was not available; the dam was presumably infected after 144 days of gestation.

A female calf showed typical malformations at the large joints of the limbs and the upper jaw and was borne dead after 281 days of gestation (infection of the mother at day 92). Unfortunately, no samples were available in this case, but its male twin showed no abnormalities, and neither antibodies nor virus was detectable.

**Figure 1:**
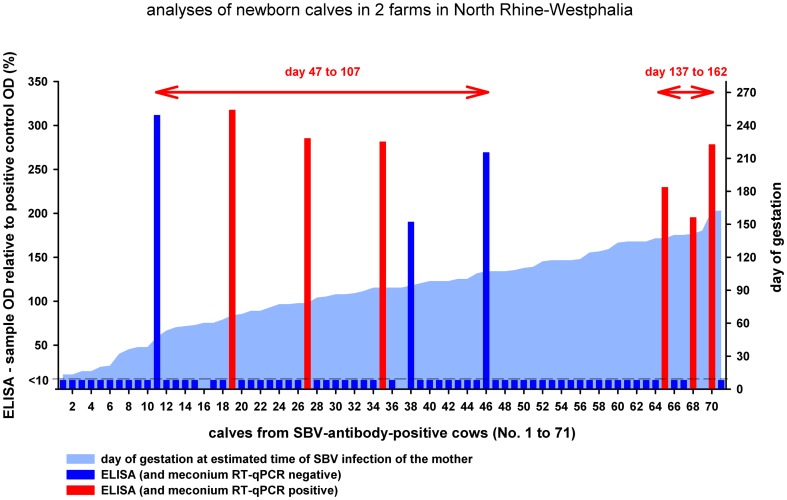
Detailed analyses of newborn calves in 2 farms in North Rhine-Westphalia. Meconium samples of calves whose ELISA results are marked in red tested positive by real-time RT-PCR; blue ELISA results correspond to negative PCR results. The day of gestation at the estimated time of SBV-infection of the respective cow is symbolized by a light blue area.

Based on the detection of viral genome or antibodies the calves borne during the study period were divided into 4 groups (table 1). 36 calves were borne after an infection of the dam between days 47 and 107 of gestation, and only in 6 serum samples taken pre-colostrally SBV-antibody titers could be detected (calves number 11, 19, 27, 35, 38, and 46; figure 1, table 1). All ELISA-scores were markedly high. Additionally, in 3 of these cases viral RNA was present in meconium swabs (calves number 19, 27, and 35). Three further calves with high pre-colostral antibody titers and SBV-genome in meconium samples were borne after an infection of their mothers between days 137 and 162 of pregnancy (calves number 65, 68, and 70; figure 1, table 1). Here in one of the 6 calves with SBV-genome detection in the meconium, viral RNA was present in serum samples as well. An infection before day 47 (n = 10) or between days 108 and 136 (n = 17) resulted neither in pre-colostral antibodies nor in detectable viral genome after birth (table 1). All calves that tested positive by real-time RT-PCR and/or ELISA were borne alive with no visible clinical signs.

## Discussion

In autumn 2011, a hitherto unknown, insect-transmitted orthobunyavirus related to AKAV was identified in blood samples of diseased German cattle. So far, SBV-specific antibodies were not detected in European livestock before 2011 [10], [12], [13], while thereafter nearly every animal seroconverted in the core region of the epidemic [3], [4], [14]. In the present study, an identically high seroprevalence among adult animals was observed, and more than 80% of the cows tested in December 2011 were seropositive. Thus, nearly every dam whose offspring was examined became infected during pregnancy; time points of gestation, however, differed among the animals.

Based on the PCR results, animals of both holdings were infected in autumn, the season with the highest activity of the insect-vectors responsible for virus transmission. As suggested by another cattle farm located in the same area, SBV spread extremely rapidly within a naive herd in that period [15]. On the aforementioned farm, blood samples were regularly taken from all cows and in only 6 out of 58 animals SBV-genome was detected, however, the seroprevalence reached 100% within 3 weeks. Accordingly, in 1 out of 8 (holding A) and 2 out of 5 (holding B) blood samples taken at the beginning of the present study viral genome was present. Consequently, all animals of holding A and B, respectively, are considered to be infected at the same time, and the calculation of the gestation day at infection based on the day of detectable viral RNA in individual blood samples.

Until now, most of the presumably SBV-caused births of dead and/or malformed lambs and calves were reported after full-term pregnancy [16], [17]. The same was observed in the present study, the only exception was an abortion after 190 days of gestation. Additionally, the birth of both, one malformed and one healthy offspring within the same litter was observed in one cow, and this is not unusual after an SBV- or AKAV-infection [18], [19]. However, SBV could not be confirmed as the causative agent in any abnormal pregnancy in the present study and viral RNA was not detectable in the fetuses and newborns. Accordingly, not all malformed fetuses suspected of SBV or AKAV infection tested positive by PCR or virus isolation in recent studies [20]–[22]. The most accepted explanation is that the virus that caused the initial lesions months before abortion, stillbirth or birth is no longer present in the newborns [7]. Hence, SBV genome is detected much more frequently in aborted, stillborn and/or malformed lambs (length of gestation in sheep approximate 5 months) than in calves (9 months) [23]. Furthermore, compared to meconium swabs used in this study, brain samples are a more suitable material [21], but sampling is complicated and only possible in dead calves.

In contrast to the aborted or stillborn calves, SBV-RNA was found in the meconium of 6 clinically health newborns, in one of them viral RNA was present in serum samples as well. Since in all pre-colostral sera of the PCR-positive fetuses high titers of SBV-specific antibodies were detected as well, the genome detection does not indicate the possibility to develop immunotolerance to and establish a persistent infection with SBV. The reason for the detection of viral RNA despite the presence of antibodies remains unclear. However, it can be speculated that both an antibody response and the detection of SBV-RNA in those fetuses is the consequence of extensive SBV-replication, which only seldom leads to abortion, malformation or stillbirth. The considerable virus replication leads to high viral load in several fetal organs and placental fluid [11] from where it potentially ends up in the meconium.

In addition to real-time PCR results, the detection of pre-colostral SBV-specific antibodies that are produced by the fetus itself once it became immunocompetent [24], is a valuable tool to confirm an SBV-infection of the fetus during pregnancy [21], [22]. Indeed, 9 out of 71 calves tested positive by ELISA, and only 6 of them also by PCR; and all of them were borne alive with no visible clinical signs. As also described for AKAV before [9], [25], the presence of SBV-specific antibodies before colostrum intake in clinically inconspicuous calves indicates that in cattle only a small proportion of infection causes the birth of malformed and/or dead animals. Moreover, not nearly every experimental inoculation with AKAV during the critical period of pregnancy lead to fetal infection resulting in abnormalities or antibody response [18]. Our results fully confirm this observation for natural infection with SBV as well. Moreover, the small amount of aborted or malformed calves related to the total number of calves borne on the monitored holding during the study period are in agreement with the low rate of stillbirth or malformation caused by fetal SBV-infection on population level (approximately 0.5% in Dutch dairy herds) [6].

From the large number of dams (n = 70) naturally infected between days 13 and 162 of pregnancy, only a limited amount gave birth to malformed or virus and/or antibody positive calves. The low number of pre-colostrally seropositive calves from infected cows can only be explained by the absence of fetal infection in most of the cases or if the fetus was infected, by the absence of an antibody response detectable by ELISA. The time point of infection of the 9 seropositive calves is also indicating that within a large time frame no immunotolerance was induced. High titers of SBV-specific antibodies could be detected even if the fetuses were most likely infected before day 90 of pregnancy, which is the well know period e.g. for the generation of BVDV immunotolerant persistently infected calves. After experimental inoculation of cows with AKAV pre-colostral antibodies were detected at the earliest in the newborn when the dam was infected at day 70 of gestation [9]. In the present study, blood samples taken from every dam during the acute phase of the disease are not available and, therefore, the exact date of infection for every animal is not known. Consequently, a possible explanation for seropositive calves whose mothers were presumably infected before day 70 may be an SBV-infection later in pregnancy.

Unfortunately, samples of calves whose mothers were infected with SBV between day 162 and the end of pregnancy were not available (calves borne between infection of the dams and the first identification of SBV); consequently, the percentage of malformed calves, abortion or seroconversion in this timeframe is unknown and should be evaluated in further studies. However, the mothers of all nine calves with high pre-colostral antibody titers were presumably infected between days 47 and 162 of gestation, and malformed or dead calves were borne after an infection between days 60 and 144 which is within the critical timeframe suggested by viruses closely related to SBV [7]–[9].
